# Deep Learning of Explainable EEG Patterns as Dynamic Spatiotemporal Clusters and Rules in a Brain-Inspired Spiking Neural Network

**DOI:** 10.3390/s21144900

**Published:** 2021-07-19

**Authors:** Maryam Doborjeh, Zohreh Doborjeh, Nikola Kasabov, Molood Barati, Grace Y. Wang

**Affiliations:** 1 School of Engineering, Computer and Mathematical Sciences, Auckland University of Technology, Auckland 1010, New Zealand; nkasabov@aut.ac.nz (N.K.); molud.barati@aut.ac.nz (M.B.); 2 Department of Audiology, Faculty of Medical and Health Sciences, School of Population Health, The University of Auckland, Auckland 1023, New Zealand; zohreh.doborjeh@auckland.ac.nz; 3 George Moore Chair of Data Analytics, School of Computing, Engineering and Intelligent Systems, Ulster University, Derry/Londonderry BT48 7JL, UK; 4 Department of Psychology and Neuroscience, Auckland University of Technology, Auckland 0627, New Zealand; grace.wang@aut.ac.nz

**Keywords:** interpretable, explainable, dynamic clustering, feature selection, spiking neural networks, spatiotemporal data, EEG data

## Abstract

The paper proposes a new method for deep learning and knowledge discovery in a brain-inspired Spiking Neural Networks (SNN) architecture that enhances the model’s explainability while learning from streaming spatiotemporal brain data (STBD) in an incremental and on-line mode of operation. This led to the extraction of spatiotemporal rules from SNN models that explain why a certain decision (output prediction) was made by the model. During the learning process, the SNN created dynamic neural clusters, captured as polygons, which evolved in time and continuously changed their size and shape. The dynamic patterns of the clusters were quantitatively analyzed to identify the important STBD features that correspond to the most activated brain regions. We studied the trend of dynamically created clusters and their spike-driven events that occur together in specific space and time. The research contributes to: (1) enhanced interpretability of SNN learning behavior through dynamic neural clustering; (2) feature selection and enhanced accuracy of classification; (3) spatiotemporal rules to support model explainability; and (4) a better understanding of the dynamics in STBD in terms of feature interaction. The clustering method was applied to a case study of Electroencephalogram (EEG) data, recorded from a healthy control group (*n* = 21) and opiate use (*n* = 18) subjects while they were performing a cognitive task. The SNN models of EEG demonstrated different trends of dynamic clusters across the groups. This suggested to select a group of marker EEG features and resulted in an improved accuracy of EEG classification to 92%, when compared with all-feature classification. During learning of EEG data, the areas of neurons in the SNN model that form adjacent clusters (corresponding to neighboring EEG channels) were detected as fuzzy boundaries that explain overlapping activity of brain regions for each group of subjects.

## 1. Introduction

Spiking Neural Networks (SNNs) are computational models of biological neurons that resemble the brain information proceeding mechanism through simulated neurons’ input and output synapses and synaptic plasticity structures [1]. SNNs are the third generation of artificial neural networks (ANN) and compared to perceptron-type neuron, they encompass the time component while accumulating the neuron’s inputs and generating temporal outputs. The literature suggests that SNNs are energy efficient and hardware friendly [2,3,4,5] compared to other artificial neural networks in machine learning (ML) systems. They have been successfully applied to various domains for classification and prediction (prognosis and diagnosis) of outcomes in temporal or spatiotemporal datasets such as classification of cognitive states using Electroencephalogram (EEG) [6,7,8,9], event-related potential (ERP) [10,11,12], and functional Magnetic Resonance Imaging (MRI) [13,14,15,16]. Several applications of SNNs are proposed in the medical domain for prognosis and diagnosis of diseases through modelling of bio-signals and biomedical images. For instance, SNN was used for modelling Alzheimer’s disease with a high accuracy of detection [17]. In clinical applications of ML, along with the accuracy of classification/prediction of health states, the ML explainability is also of crucial importance. This refers to the degree to which an end-user (clinical practitioner) comprehends the reason of a certain decision (classifier outcome). Although SNNs have shown reasonable performance in the modelling of spatiotemporal brain data (STBD), they remain as black boxes where the interpretation of the trained SNN models is yet limited. Therefore, new methods are required for extracting the knowledge stored in a spiking neuron and their internal time-varying weights that allow to explain the model output decisions. The proposed brain-inspired SNN (BI-SNN) architecture NeuCube [18] allowed now to “open the black box” and even to extract spatiotemporal rules [19,20].

In our previous study [21], a method for dynamic clustering in SNN was proposed as a procedure of grouping neurons with respect to their spatiotemporal activities produced while learning from streaming input data. This initiated the concept of explainability and interpretability in SNN models’ learning behavior. In the current study, we applied the dynamic clustering technique to differentiate SNN models while learning from multiple classes of streaming STBD. Then, we extracted the information stored in the SNN models (dynamics of spiking activity and connection weights) and proposed new methods to improve the model accuracy as well as explainability. The main two outcomes of the current research are as follows:

- Detecting informative spatiotemporal variables with respect to the dynamic evolving spike-driven patterns during the learning process in SNN models. This resulted in improving the output prediction/classification accuracy.
- Extracting spatiotemporal rules of spike occurrence during the dynamic clustering, which enhanced the interpretability and explainability of SNN learning behavior.

The current paper is organized as follows: Section 2 presents a methodology that includes methods for dynamic spatiotemporal clustering, feature selection, validity measurement, and spatiotemporal Fuzzy clusters and rule extraction in SNN models; Section 3 applies the proposed methods to a case study of EEG and demonstrates the results of the clustering approach; and finally, Section 4 presents the research conclusion and future direction.

## 2. Materials and Methods

### 2.1. Method for Dynamic Spatiotemporal Clustering of Streaming Data in Spiking Neural Networks

This section proposes a methodology for extraction of knowledge from a BI-SNN that combines different computational methods in a pipeline as follows:

1. Spatiotemporal data encoding.
2. SNN mapping and initializing.
3. Unsupervised learning in SNN and simultaneously clustering the neurons.
4. Quantitative analysis of the dynamic clustering patterns.
5. Spatiotemporal fuzzy clustering.
6. Spatiotemporal rule extraction from SNN clustering patterns.
7. Supervised learning and pattern classification.

The above steps are further elaborated in the following sections.

First, a dynamic clustering is applied to the BI-SNN model for clustering the neurons with respect to the similarity in their spiking activities, evoked during an incremental learning procedure with streaming STBD. Then, the generated spike-driven events in the BI-SNN model are visualized and analyzed for exacting spatiotemporal rules that allowed the SNN outputs (classification) to be better explained and the brain data to be interpreted. The applied clustering method builds upon our previous research in [21]. Our proposed methodology includes the following procedures: 

Data encoding: spatiotemporal data streams are encoded into spikes, which are binary values of 1 and −1 referring, respectively, to upward and downward changes in the temporal brain data over time. Here, a threshold-dependent encoding method is employed to generate positive (excitatory) and negative (inhibitory) spikes in certain time t; hence, the dynamics of the data are preserved. Thus far, a variety of encoding algorithms were developed, among which some popular methods are: temporal encoding [13,22,23], Ben’s Spikes Algorithm (BSA) [24] and Population Rank Coding [25].

Data mapping: a 3-dimensional BI-SNN model is mapped that topologically preserves the spatial information of brain data variables. Here, a brain atlas, called Talairach [26,27], is used for mapping the brain EEG data into the BI-SNN models [18].

SNN model initialization: the SNN connection weights are initially established with the use of small-world connectivity rule [18] which is inspired by biological systems [28,29]. The computational model of the spiking neurons is Leaky Integrated-and-Fire (LIF) [30]. In this model, the membrane potential v(t) of a neuron increases with every input spike at a time *t*, multiplied by the synaptic efficacy (strength), until it reaches a certain firing threshold θ. The potential, however, decreases between the sequential spikes by the leak parameter. When the firing threshold is reached, an output spike is emitted, and the membrane potential is reset to an initial state. The LIF model is mathematically defined as follow:

(1) $$\tau_{m}\frac{dv}{dt}=v_{rest}-v\left(t\right)+RI\left(t\right)$$

where $\tau_{m}$ is the membrane time constant,$\;v_{rest}$ is the resting potential, *I* and *R* are the input current and the resistance, respectively.

Unsupervised learning and dynamic clustering: SNN models learn from the spatiotemporal interactions between the brain data variables and the model connectivity and spiking activity are incrementally clustered. Here, the biologically plausible Spike-Timing-Dependent Plasticity (STDP) learning rule [31] is employed to learn the spatiotemporal patterns of input data streams. Throughout the learning procedure, the SNN connections weights are adapted, and the neurons are clustered in a continuous and incremental mode with respect to their spiking activity evoked by different input neurons (cluster centers). STDP is an example of Hebbian learning rule which depends on the relative timing of pre- and postsynaptic action potentials, defined using the following relation:

(2) $$F\left(∆t\right)=\{\begin{array}{c} \;A_{+}\exp\left(∆t/\tau_{+}\right)\;if\;∆t<0\;\\ -A_{-}\exp\left(-∆t/\tau_{-}\right)\;if\;∆t\geq 0 \end{array}\;$$

where F(∆t) defines the synaptic modification elicited from a single pair of pre- and postsynaptic spikes separated by a time interval $∆t=t_{pre}-t_{post}$. The parameters *A*_+_ and *A*_−_ define the maximum quantities of synaptic modification, which transpire when ∆t ≈ 0. The parameters $\tau_{+}$ and $\tau_{-}$ determine the ranges of pre-to-post-synaptic inter spike intervals over which the synaptic strengthening and weakening occurs.

**Figure 1:**
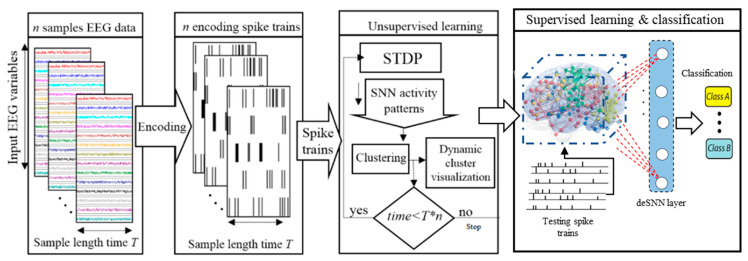
A block diagram of the clustering of neurons in BI-SNN architecture during STDP learning and the SNN pattern classification.

The main objective of the dynamic clustering approach is knowledge discovery in the BI-SNN models by detecting the associated spatiotemporal patterns of changes (while streaming input data), which are dynamically adapted through learning with respect to the interactions between input neurons (brain data variables). This clustering is based on unsupervised STDP learning that results in an improved interpretation and explainability of the interactions between the data variables. The procedure of dynamic spatiotemporal clustering in BI-SNN models is graphically shown in Figure 1.

For this dynamic clustering, the cluster centers are defined in advance according to the spatial positions of the brain data variables (e.g., EEG electrodes) which are mapped as input neurons into the BI-SNN model. Then, during the STDP learning process, the input brain data are streaming via the input neurons (clusters centers) and trigger the transmission of spikes between the neurons. The greater number of spikes exchanged between a pair of neurons i and j, the greater the connection weight ($w_{ij})$ becomes between them, where $w_{ij}$ denotes the weight specifying the connection strength. Throughout the clustering process, every neuron in the SNN model can be assigned to different clusters with different membership values. This membership is defined according to the number of spikes that a neuron receives from each of the clusters’ centers (input neurons which map the brain data variables, such as EEG electrodes). A neuron is assigned to a cluster if it receives the greatest number of spikes from this cluster center compared to other centers.

In the BI-SNN model with N neurons, the input neurons are assigned to the cluster centres and taken by the input data variables, while the rest of the neurons are unlabeled. The objective is to assign the cluster labels to the unlabeled neurons in the BI-SNN model. To this end, we used the concept of spreading activation in network theory from [32] and performed as follows:

The neurons in the SNN model are indexed from 1 to N ascendingly with respect to the order of their spatial (x, y, z) coordinates. The input neurons are marked as the information source and defined using an N×v matrix $F_{src}$ in which F_src (i,j)=1 if neuron i is the input neuron for variable j; otherwise $F_{src}\left(i,j\right)=0$, where N is the number of neurons in the BI-SNN model and v is the number of input data variables (e.g., EEG variables). While streaming spatiotemporal data, each neuron in the BI-SNN model receives a different ratio of information from different input variables. The ratio of the received information can be computed through the following procedure:

An affinity N×N matrix A is defined on the SNN model that displays the sum of the spikes that are exchanged between neurons i and j (i=1,…, N and j=1,…,N) via connection $w_{ij}$. The amount of information that is exchanged between the neurons is computed as follows:

(3) $$\begin{array}{c} {A^{\prime }}_{ij}=A_{ij}+A_{ji}\;i\neq j\\ {A^{\prime }}_{ij}=0\;i=j \end{array}$$

where the element $A_{ij\;}$ displays the number of spikes transmitted from neuron i to j, while $A_{ji\;}$ indicates the number of spikes transmitted from neuron j to i. Since a neuron does not send a spike to itself, the entry for $A{\;}_{ij}$ is 0 when i=j.

(4) $$T_{i}=\overset{N}{\underset{j=1}{\sum }}A\prime_{ij\;}\;i=1\;to\;N$$

Thus, $T_{i}$ is the sum of the elements in the ith row of matrix $A^{\prime }$. Then, the affinity matrix A is normalized using S=D A D, where D is an N×N diagonal matrix, where its (i,i)-element is defined by $D_{ii}=\left(\frac{1}{\sqrt{T_{i}}}\right)$ and S is an N×N normalized matrix that encodes the spike propagation in the SNN model.

Iterate the below equation until it converges, where α parameter is in the (0, 1) range.

(5) $$F\left(t+1\right)=\alpha SF\left(t\right)+\left(1-\alpha \right)F_{src}$$

The limit of F(t) is denoted by $\;F^{*}$ and defined as follow, where I is an identity matrix and the output $F^{*}$ has N rows (representing all neurons in the SNN model) and v columns (representing the input variables).

(6) $$\;F^{*}=\underset{t\to \infty }{\lim}F\left(t\right)={\left(I-\alpha S\right)}^{-1}\;F_{src}$$

The element $\;F^{*}{}_{ij}$ represents the relative information amount that a neuron i in the BI-SNN model receives from an input neuron j. By computing the $\;arg\;{max}_{j=1,\ldots v}\;F^{*}{}_{ij}$, the neurons in the SNN model are classified into different input variables. This results in clustering the neurons into v inputs. This procedure can be better understood as follows:

In an SNN model, the input information is propagated from input neurons (sources of information) to other neurons. At the beginning of the STDP learning in the SNN model, only the input neurons (centroids of the clusters) have received the information ($F^{*}=F_{src})$. When the learning procedure increments with sets of spatiotemporal streams over time, the other neurons will also receive a ratio of information from one or more input neurons. Therefore, neurons are being clustered with respect to the amount of information that they receive from each of the inputs. In such a way, neural clusters are created and evolved over time in an incremental way during STDP learning.

The dynamic visualization of the clusters illustrates the time points in which the clusters are generated, and it shows how the clusters are altered over time. Such clusters are formed in a 3-dimensional view and have different size and shapes. The size and the creation-time of a cluster signifies the importance of the cluster center in the trained SNN model, and consequently, the importance of the corresponding input variable in the data. The proposed clustering algorithm is given in Algorithm 1.
**Algorithm 1.** The dynamic spatiotemporal clustering algorithm at time point *t* of the unsupervised learning process.
**Input:** Input spike data sp, number of neurons in the SNN model N, number of input variables v, connection weights w[N,N], and parameter α, PSP, STDP, time t **Output**: A vector of labelled neurons k, vector of spik events for each cluster 1: **Procedure**
2: [L V]=size(sp) 3: $Fsrc\;\in \;R^{N\times v}$**,**
$A\in \;R^{N\times N}$ 4: For each time point *t* from the input stream data Do 5: Update w with STDP 6: S=D A D 7: $F^{*}={\left(I-\alpha S\right)}^{-1}\;F_{src}$ 8: $k=arg\;max_{j=1,\ldots v}\;F^{*}{}_{ij}$ 9: Visualization of the clusters 10: Spatiotemporal rules within each cluster Do 11: If PSP(t)≥event−threshold 12: Cluster fires as active event in time t. 13: End if 14: End for 15: Algorithms to generate a set of spatiotemporal rules 16: **End of procedure**

### 2.2. SNN Model Explainability through Dynamic Clustering Method

**Figure 2:**
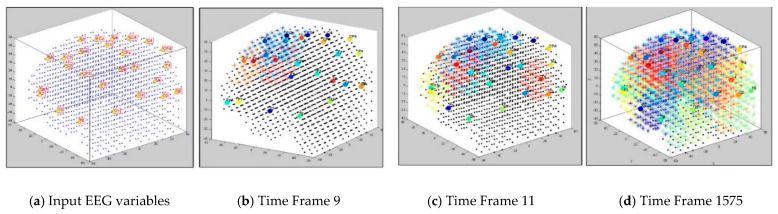
Four steps visualization of dynamic clustering in a BI-SNN model, corresponding to 26 EEG channels (recorded from 21 control subjects) during unsupervised STDP learning. The total number of time frames is 21 samples × 75 EEG time points = 1575 data points.

The dynamics of the cluster creation can be scrutinized to explore the “hidden” spatiotemporal learning patterns in SNN to enhance the explainability of the model while learning from streaming data. In this study, we illustrate the proposed method on EEG data recorded from 26 scalp electrodes whilst two groups of participants (healthy control group, and opiate addiction group (OP)) performed an inhibition-related cognitive task (called GO-NOGO). This EEG data was previously analyzed in [33]. Figure 2 shows an exemplar visualization of the dynamic clustering in the BI-SNN model while learning from input EEG data streams. This illustrates that a BI-SNN model was initially mapped using a brain template (e.g., Talairach [26,27]) and the 26 EEG electrodes were assigned as input neurons (cluster centers). Then the BI-SNN was incrementally clustered by different centers during the STDP learning with EEG samples. Based on the LIF computational model [30] of the spiking neurons in BI-SNN, the neuron’s postsynaptic potential (PSP) enhances when a new input spike arrives in the neuron. When the PSP(t) surpasses a firing threshold at time t, the neuron releases an output spike and sends it to the rest of the neurons connected to it. This process controls the spiking activity of the neurons, while the STDP learning adapts their internal connection weights.

While dynamic clusters are created in SNN during the STDP learning process (an example is shown in Figure 2), significant dynamic patterns were associated with each cluster as follows:

- Input spike train $\left(s_{t}\right)$ to an SNN model.
- The mean of the cluster’s postsynaptic potentials PSP, indicated by $\mu_{\mathrm{PSP}\left(t\right)}$.
- The mean of the cluster’s spiking rates, indicated by $sr_{t}$.
- The size of the cluster (number of neurons).
- The mean of the neuron’s memberships (the number of spikes received by neurons from the cluster center).

**Figure 3:**
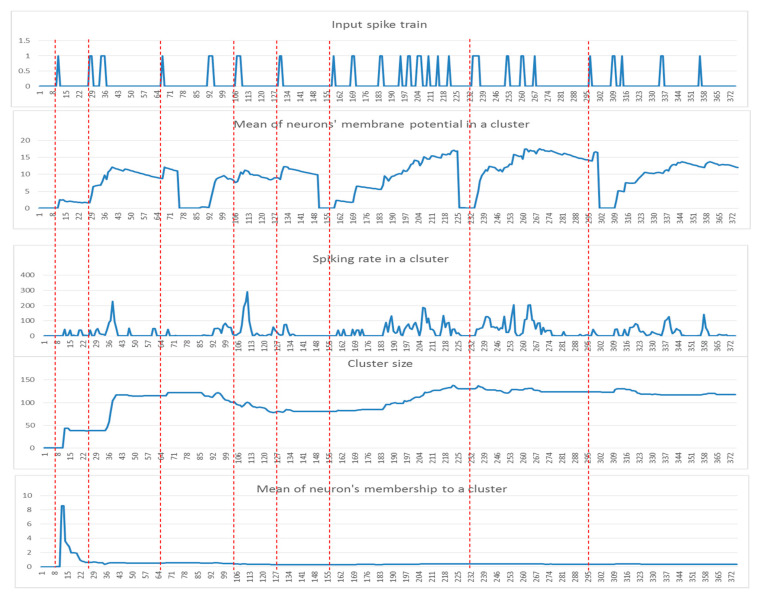
Examples of the five dynamic patterns:$\;\left(s_{t}\right)$, ($\mu_{PSP\left(t\right)})$, ($sr_{t}$), the cluster size, and the neurons memberships of one cluster (for EEG channel T4) corresponding to a time-window of 75 time points for five samples from the control group.

These patterns can be used to detect informative spatiotemporal EEG variables that demonstrate significant discrimination between samples from different classes (e.g., control and OP). In Figure 3, examples of these five dynamic patterns (from one randomly selected EEG variable in Figure 2) are shown.

- Among these five patterns of the cluster evolution, we further investigated the PSP(t) patterns using the following techniques:
- Local maximum $P_{max}\left(t\right):$ the maximum value of the PSP(t) was measured for each data sample.
- The area under a curve: this is computed from the PSP(t) of each data sample defined by $\int_{1}^{l}P\left(t\right)dt,$ where l is the length of each sample (time points).
- Mid of potential: this is an average of the min value and max value in the PSP(t), measured through (max+min) /2.

### 2.3. Spatiotemporal Fuzzy Clusters in SNN Models

Hitherto, the paper presented that every cluster in the BI-SNN evolves dynamically during the STDP learning. At each time point t of the STDP, every cluster is demonstrated as a crisp cluster which means its members (neurons) belong only to one cluster center at each time t and no neuron is shared between the clusters. However, in the next time point of the STDP learning, a cluster may lose some of its members (neurons) and scale down or it may involve more neurons and scale up in size. Therefore, some neurons that belonged to a certain cluster at the previous state of the network may move to a new cluster at the current state and keep exchanging between the clusters in the following timepoints. When the STDP learning is completed, those neurons that were exchanged between the adjacent clusters during the learning process were identified as the shared spatial areas of neurons (boundaries) between the clusters (brain regions). Any pair of clusters that have wider boundary of the shared neurons suggest a stronger spatiotemporal interaction over time. This is experimentally illustrated in Section 3.3.

### 2.4. Enhancing the SNN Explainability through Spatiotemporal Spike Rule Extraction

During the dynamic spatiotemporal clustering in SNN, the clusters are evolving in time. Here, a spatiotemporal rule extraction method is proposed to detect specific patterns of spatiotemporal spike events occurred inside the clusters at a specific space and time. This led to define different spatiotemporal rules $R_{j=\left\{1,2,\ldots ,k\right\}}$ for the SNN models trained with different classes of data, where k is the number of classes (in this case, 2 classes: control and OP). The spatiotemporal rules are described with respect to the spike events that occurred in spatial locations (cluster c={1,…, l)) at certain times. Each spatial location is defined as a cluster of spiking neurons and acts as a binary unit depending on its activation level. The level of activation for each cluster is identified by a spike-emitting-threshold ℓ, applied to the PSP patterns (demonstrated in Section 3.4). If the PSP pattern of cluster c at time t exceeds the ℓ threshold, then this cluster is recognized as an active cluster that produces a spike at t. The spike-event sequence of each cluster c at time t is denoted by $c_{i}\left(t\right)$ and described as follows:

(7) $$c_{i}\left(t\right)=\{\begin{array}{c} 1\;PSP\left(t\right)\geq \ell \\ 0\;otherwise\; \end{array},\;t=1:T,\;i=1:l\;$$

where T is the temporal length of PSP pattern of each cluster c, while l refers the number of clusters (in this case, the number of EEG variables).

A spatiotemporal rule $R_{i}$ shows a trajectory of set of actions (denoted by A) from the $c_{i}\left(t\right)$ that occurred at different spatial positions and times. An action A happens in cluster c when there is a series of spike events ($c_{i}\left(ℒ\right)>0$) that occurred sequentially during a specific time-interval ℒ and is associated with an order of time ord. This means multiple actions can occur in the same spatial location, but with different time orders. An action A and a symbolic representation of the rule $R_{i}$ are described as follows:

(8) $$A=<c_{i}\left(ℒ\right)>0\;,\;ord>$$

(9) $$R_{i}=IF\;A_{1}\;AND\;A_{2}\;AND\ldots AND\;A_{n}\;THEN\;Output=output_{j}$$

The procedure for detecting the temporal orders in which spike actions occurred in each cluster is demonstrated in Algorithm 2.
**Algorithm 2.** Defining the order of the time interval when spike actions A are detected.**Inputs**: Cluster c, Number of clusters l, PSP timeseries, PSP temporal length T, Spike-events in clusters $c_{i}\left(t\right)$ and spike time-interval ℒ **Outputs**: Rules R=(A, ord) as set of Action A and time orders **Procedure:** For c=1 to l //for all the clusters Baseline ←1
While (Baseline<T−ℒ)
If ($\mathrm{Length}\;\mathrm{of}\;\{c_{i}\left(\mathrm{Baseline}:\;Baseline+ℒ\right)>0\}\;\mathrm{equal}\;\mathrm{to}\;ℒ)$ //sequential ℒ number of spikes Action (c, Baseline) ←A
End If
Baseline←Baseline+1 End while End For Print sets of Actions as Rules For c = 1 to l *Ord*←1 For *t = 1 to T* If Actions(c,t)=A R(ord) ←Actions(c,t) *Ord*←ord+1 End For End For
**End of Procedure**

### 2.5. Validity Measurement of the SNN Clustering

**Figure 4:**
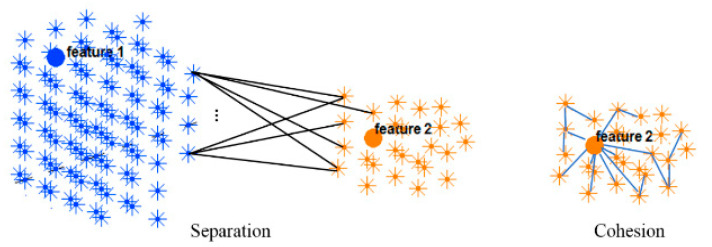
Two clusters of neurons in an SNN model were generated, each of which was associated with one EEG variable acting as a cluster center (input feature allocated to an input neuron). Cohesion measures how related the neurons are in a cluster through averaging the connection weights in the cluster, while separation measures how distinct a cluster is from other clusters through averaging the connection weights between the clusters.

This section evaluates the dynamic spatiotemporal clustering through measuring how a cluster’s member (neuron) fits into its own cluster compared to other clusters. Since there was no class label information at the STDP unsupervised learning phase in the SNN model, here we employed an internal measurement technique, called silhouette coefficient validity method. This validity measurement is based on the “cohesion and separation” concept [34,35] graphically shown in Figure 4 for two adjacent clusters extracted from the SNN models from Figure 3.

Cohesion measures how similar the members (neurons in this case) are within a cluster, whereas separation defines how distinctive and well-separated a cluster is from other clusters. For clustering validation, the objective is to maximize the cohesion metric while minimizing the separation metric. Here, the cluster cohesion is defined with respect to the average of the connection weights between the internal neurons of a cluster in the SNN model. On the other hand, the average of the connection weights between neurons of a cluster and neurons of a neighboring cluster describes the cluster separation. A neuronal cluster in an SNN model is valid if its cohesion metric is higher than the total of all the separation metric within its neighborhood.

The silhouette validates the homogeneity within clusters through including both cohesion and separation to assess how close a neuron is to its own cluster center (cohesion) compared to other clusters (separation). For each neuron i within a cluster, value x(i) is the average cohesion of i to all other neurons in the same cluster. It shows how well i is assigned to its own cluster, so that a larger value refers to a more appropriate assignment. On the other hand, value y(i) is the average separation between a neuron i and other neurons in a neighboring cluster.

(10) $$s\left(i\right)=\frac{x\left(i\right)-y\left(i\right)}{max\left\{y\left(i\right),x\left(i\right)\right\}}\;\;$$

**Figure 5:**
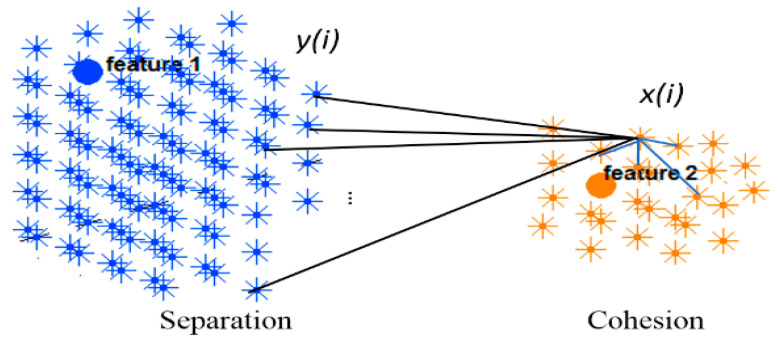
Silhouette method exemplified on two clusters.

The silhouette value is agreed to be in an interval of −1≤s(i)≤1, and a value closer to 1 implies that the neuron is well-matched to its own cluster. If most of the neurons have a high silhouette value, then the clustering configuration is valid. Figure 5 shows the silhouette method exemplified using two adjacent clusters. In an SNN model with N number of spiking neurons and a set of input neurons γ={1,…,c}, the clustering method is performed on a normalized affinity matrix which encoded the N × N information of the SNN connection weights. Through the clustering, every neuron i is clustered into an input neuron γ (cluster centre) with respect to the propagation number of spikes which is relative to the connection weight between neuron i and the center γ. The $F_{i\gamma }$ reveals the relative number of spikes that a neuron i receives from each input neuron γ and it defines the membership value of i to each cluster centre. Within a cluster, when neuron i is connected to m neurons, the average of the connection weights between i and all m neurons define the cohesion of i to its cluster. This cohesion is multiplied by the membership value of neuron i to its cluster center as follows:

(11) $$x\left(i\right)=\frac{\sum_{j=1}^{m}w_{ij}}{m}\times F_{i\gamma }\;\;$$

In contrast, value y(i) is the average separation between neuron *i* and k numbers of connected neurons from the f numbers of neighboring clusters as follows:

(12) $$y\left(i\right)=\frac{\sum_{n=1}^{f}\frac{\sum_{j=1}^{k}w_{ij}}{k}\times F_{i\gamma }}{f},\;\gamma =f\;$$

## 3. Results: Dynamic SNN Clustering of EEG Data, Spatiotemporal Rule Extraction and Feature Selection

The spatiotemporal clustering was applied to an EEG dataset that was recorded using a QuickCap (Neuroscan 4.3). The 26 electrodes include Fp1, Fp2, Fz, F3, F4, F7, F8, Cz, C3, C4, CP3, CPz, CP4, FC3, FCz, FC4, T3, T4, T5, T6, Pz, P3, P4, O1, O2, and Oz (10–20 International System). EEG data were recorded at the University of Auckland, New Zealand and the ethical approval was granted by the “Northern X Regional Ethics Committee of New Zealand”. The informed consent was given by all participants. Horizontal eye movements were recorded with electrodes placed 1.5 cm laterally to the outer canthus of each eye. Vertical eye movements were recorded with electrodes placed 3 mm above the middle of the left eyebrow and 1.5 cm below the middle of the left bottom eyelid. EEG data were screened visually for artifacts (Artifacts are signals recorded by EEG but not generated by the brain), normal variants and changes in alertness (the technician screening these data was blinded to group status). To reduce muscle artefacts in the EEG signal, the participants were instructed to assume a comfortable position and avoid movement during recording. Electrical impedance was always <5 KΩ. During the recording process, participants were asked to complete a cognitive task called GO-NOGO [33]. The EEG data recorded from 21 healthy control subjects and 18 opiate users (OP) were used in the present experiment.

### 3.1. Dynamic Spatiotemporal Clustering in SNN while Streaming EEG Data

**Figure 6:**
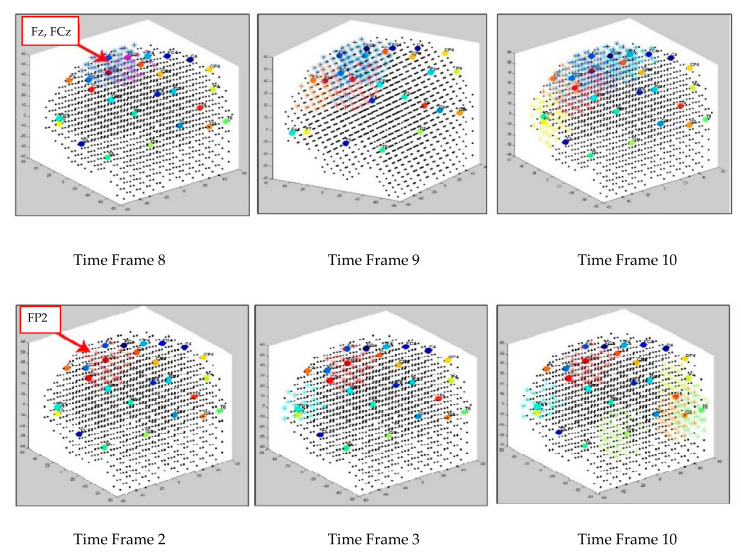
Three snapshots of the dynamic cluster creation process over time during the STDP learning in the SNN models of control (in the **upper row**) and OP (in the **lower row**).

**Figure 7:**
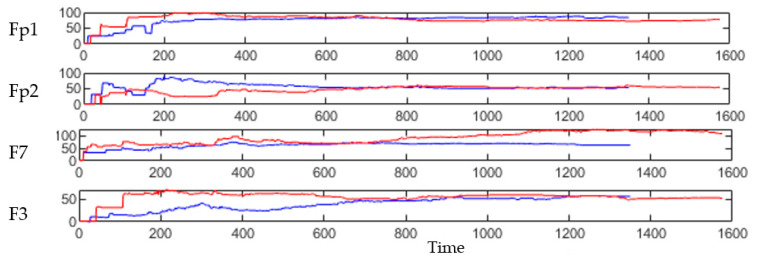
Examples of four clusters’ size changing during the STDP learning in SNN model of 21 control subjects (shown in red, in total 1575 time points were entered and trained in the model) and 18 OP subjects (shown in blue, in total 1350 time points were entered and trained in the model).

Figure 6 illustrates the creation of dynamic clusters over time while two separate SNN models are learning from the input EEG data streams of control and OP groups, respectively. The clustering procedure is started from initial SNN models (Figure 6 left cubes), where the input neurons are assigned to the EEG electrodes (cluster centers) for transmitting the input spikes into the models. Then, the SNN models were evolved dynamically, every time a new EEG data time point was entered to the SNN models for learning. In Figure 6, an example of only three timeframes of the cluster’s evolution is visualized; however, the cluster procedure was continued for the whole EEG time intervals. Here, the spatiotemporal clusters were formed and updated with every new input EEG time point entered, frame by frame. The reason that different time frames are visualized in Figure 6 is due to the time differences in cluster creation across the subject groups with respect to their EEG data. Once new clusters appeared during unsupervised STDP learning, a new frame of the clustered SNN was captured to display the stepwise changes in the cluster evolution. Figure 7 reports how the size of the clusters in SNN models of control and OP groups changed during the STDP learning with the whole-time interval of EEG data.

### 3.2. Feature Selection through Modelling Dynamic Clustering Patterns in SNN

**Figure 8:**
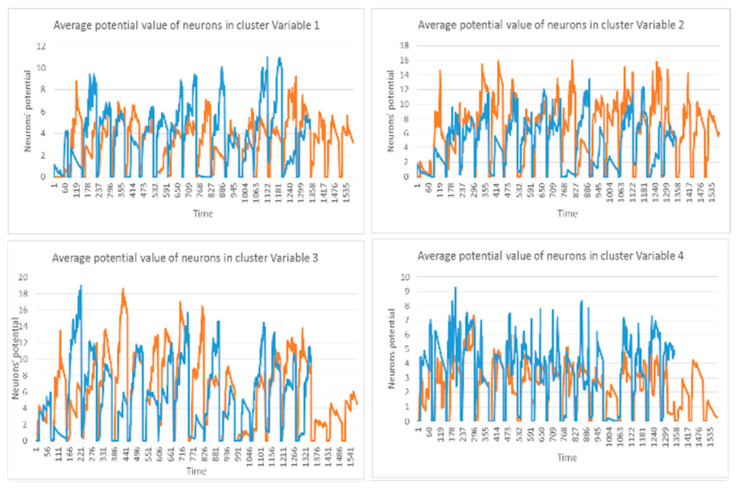
The dynamic patterns of the mean of PSP rates (an example of four clusters corresponding to Fp1, Fp2, F7, and F3 variables) during the learning process with EEG samples from the control class (in red) and OP class (in blue).

**Figure 9:**
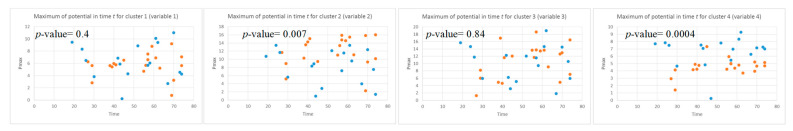
The local maximum of the potential $P_{max}\left(t\right)$ for four clusters (corresponding to Fp1, Fp2, F7, and F3 variables) that are plotted as dots in time t for all the EEG samples in two classes: control class (red) and OP class (blue). The $P_{max}\left(t\right)$ values can show the level of difference between the two classes (control and OP) in the EEG variables with p-value < 0.05 (measured by a *t*-test). The EEG variables with high *p*-value are not statistically significant.

**Figure 10:**
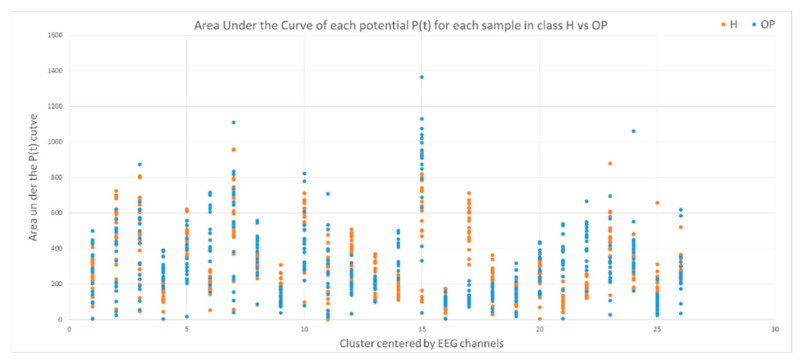
The area under curve of PSP rates for 26 clusters for all the samples in the control class (red) and OP class (blue). Discriminative patterns between class control and class OP have been observed in EEG variables with small p-value (measured by a *t*-test).

**Figure 11:**
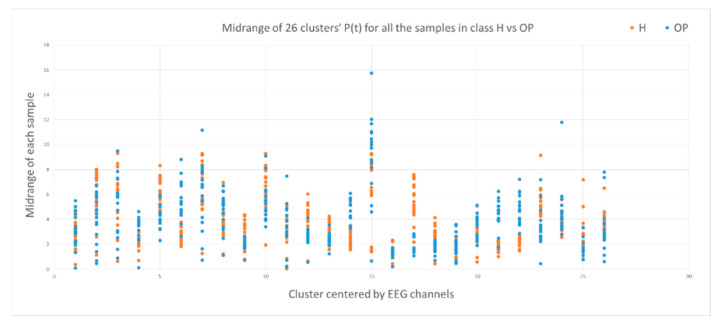
The midrange of the PSP rates corresponding to 26 clusters for all samples in control class (red) and OP class (blue). The midrange values show discriminative patterns between samples that belong to class control versus samples that belong to class OP in variables with small p-value (measured by a *t*-test).

This section illustrates the explainability of the SNN models and investigates the knowledge stored in the SNN models through analyzing the trends of clusters creation. The PSP(t) time series were analyzed to reveal how the SNN-based dynamic clustering could be useful to discriminate the EEG data samples across different classes. Here, the dynamic PSP(t) patterns were captured for all the 26 clusters during the STDP learning process in SNN models with EEG data of two classes of participants (control subjects and opiate addicts). Figure 8 depicts an example of dynamic PSP(t) visualization for only 10 clusters (related to 10 EEG electrode) in control and OP groups. These PST patterns were investigated through computing the peak of potential ($\;P_{max}\left(t\right)$) (shown in Figure 9), area under curve (Figure 10), and midrange of potential (Figure 11). Figure 9 shows that for each EEG sample, the peak of potential ($\;P_{max}\left(t\right)$) is plotted as a dot at time t. This potentially separates the samples across the classes with different degree of discrimination in the EEG features with t−value>0.05.

To identify how the dynamic clusters reveal significant differences between the classes (control and OP), a statistical *t*-test measure was applied to the plots in Figure 9, Figure 10 and Figure 11. The *t*-test results are reported in Table 1, where the mutual top 8 EEG variables refer to the potential discriminative variables to precise EEG samples to the control class and the OP class. These variables are 17, 14, 21, 22, 6, 12, 5 and 23 which, respectively, correspond to EEG electrodes CPz, C4, P4, Pz, F4, C3, T6, and Fz. Then, a SNN-based classification experiment was designed to classify the EEG samples to control and OP groups when using these top 8 variables.

The classification task is based on dynamic evolving SNN [36] classifier (deSNN) and leave-one-out cross validation method. To this end, after the unsupervised STDP learning was completed, a supervised learning was conducted to learn the relationships between the class labels and the training EEG samples. For every EEG sample that was used previously for unsupervised learning in the BI-SNN, one neuron is created on the output layer and connected to the neurons in the trained model. The connections between the SNN neurons and output layer neurons are initialized using the rank-order rule [37]. After establishing the initial connection weights, the same EEG data that were used at unsupervised learning phase are used to train the SNN mode at a supervised mode. The neuron post-synaptic potential PSP of neuron *j* at time *t* connected to neuron i in the SNN space, is calculated as follows:

(13) $$PSP\left(j,t\right)=\sum^{\;}mod^{order\left(i\right)}W_{ij}$$

where *mod* is a modulation factor (a parameter between 0 and 1) and *order(i)* is the time order of the following spikes to the connection between neurons *i* and *j*. Through this learning rule, the first spike that arrives at the output neuron *j* will have the highest value. Then, the connection weight $W_{ij}$ will be further modified according to the spike-driven synaptic plasticity learning rule using a drift parameter, which is used to modify $W_{ij}$ to take into account the occurrence of the following spikes at neuron *j* at time *t,* denoted by $\;spike_{j}\left(t\right)$*,* i.e., if there is a spike arriving from neuron *i* at time *t* after the first one was emitted, the connection weight increases by a small drift value; otherwise, it decreases by drift.

Then the trained SNN model is tested with every EEG sample to classify the individuals into OP and control groups. We performed a comparative analysis by classifying the EEG data using conventional ML methods including Support Vector Machine (SVM), Multilayer Perceptron (MLP), Multilayer Regression (MLR) and Evolving Clustering Method (ECM). Table 2 reports that the accuracy of classification is higher when using the top eight EEG features than all the 26 variables, in all the experiments [33].

**Figure 12:**
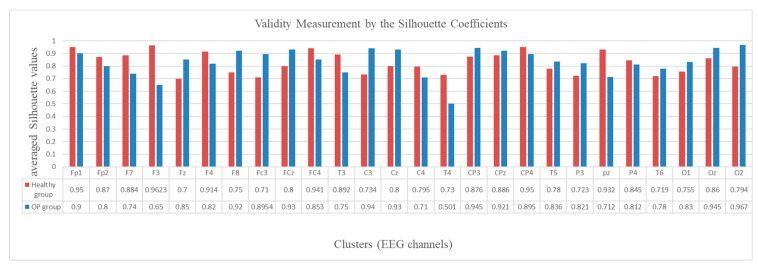
Validity measurement of the clusters generated in the SNN models of EEG data with 26 channels from the healthy control (red bar) and OP group (blue bar). The silhouette value was measured for every neuron in a cluster. Then the silhouette values were averaged over all the neurons in a cluster and represented as a validity metric for this cluster.

To evaluate the validity of the created clusters, the average of the silhouette coefficients (Equation (10)) was measured in every cluster, as shown in Figure 12. The graph shows that all the average silhouette values are positive and very close to 1, which represents a high goodness value for the clusters.

### 3.3. Spatiotemporal Fuzzy Clusters in SNN Models of EEG from Control and OP Groups

**Figure 13:**
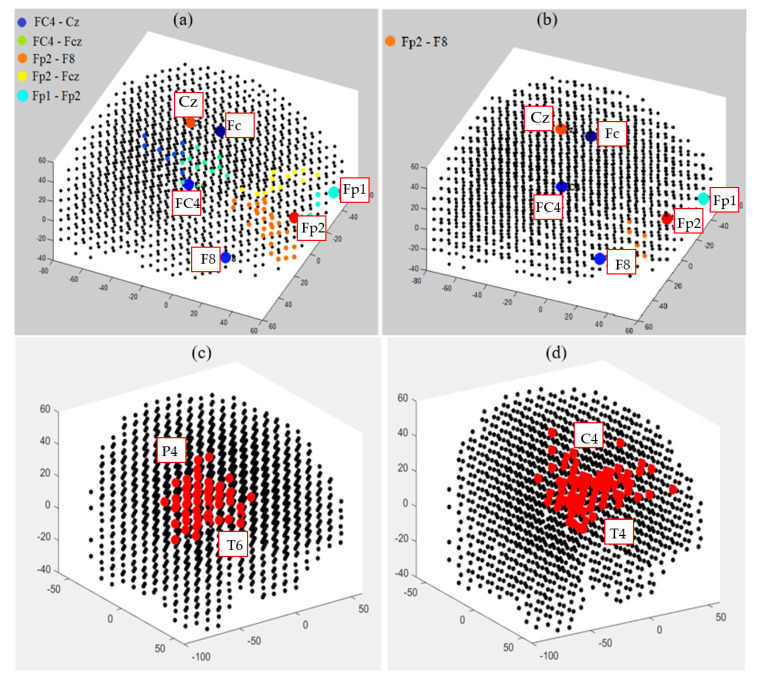
The fuzzy neural clusters (shared boundaries between clusters) captured after the unsupervised STDP learning in SNN models of (**a**) control group and (**b**) OP group. (**c**,**d**) The biggest fuzzy cluster in the control group has a size of 59 neurons, generated between P4 and T6 channels, while the biggest fuzzy cluster in OP group has a size of 70 neurons, generated between C4 and T4 channels.

This section illustrates fuzzy clusters in BI-SNN that led to improvement of the explainability of the trained models with different classes. This is to demonstrate how different neural clusters in the BI-SNN model were interacting during the STDP learning with EEG data of control vs. OP groups. Here, we detected those neurons that changed their membership between clusters at different time points of the STDP learning. These areas are fuzzy clusters that include neurons which changed their membership from one cluster to another cluster over time based on their updated membership values. It represents a notion of functional interactions between EEG electrodes across the groups. Figure 13 visualizes the areas of shared neurons between five pairs of randomly selected EEG channels. These boundaries show the intersection areas between every two adjacent crisp clusters (centered by EEG variables), shown as fuzzy clusters. Detection of these boundaries allows new knowledge to be discovered from the SNN learning patterns and enhances the model explainability, so that an end-user can better interpret the spatiotemporal interactions between EEG variables that resulted in classifying EEG samples to control or OP groups. Therefore, the decision made by the SNN models can be explained and interpreted. For example, it can be seen from Figure 13b that for the OP group, the only shared area of neurons among these five EEG channels is observed between Fp2 and F8 channels and this boundary is significantly smaller than the captured boundaries in control subjects.

### 3.4. Capturing Spatiotemporal Spike Events during Unsupervised Learning in SNN Models

Thus far, we demonstrated that the BI-SNN models of EEG data created dynamic clusters as polygons, which evolved in time and continuously changed their size and shape. In this section, we further analyzed the patterns of dynamic clusters to discover rules for spatiotemporal spike events that occurred together in both space and time during the cluster’s creation for different classes (control vs. OP groups).

**Figure 14:**
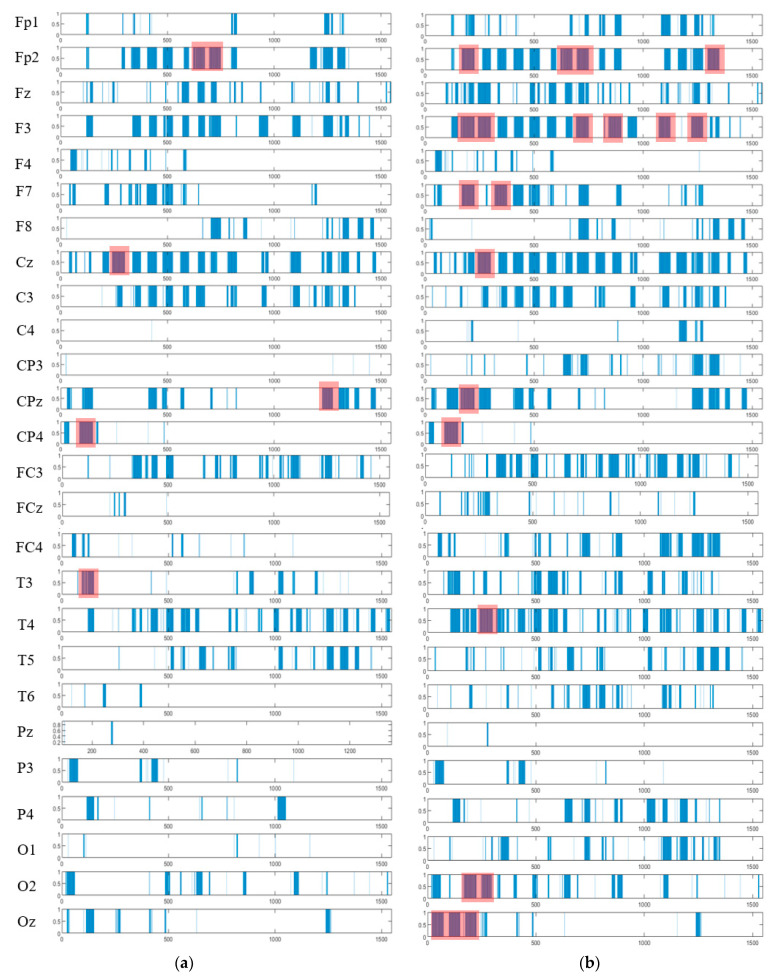
The spatiotemporal spike events (shown in blue bars) are extracted from the PSP patterns (shown in Figure 8) to demonstrate (when) and (where) the neural spike events (denoted as action A) occurred in different groups (control in (**a**) and OP in (**b**)). These spikes are events that occurred at different spatial brain regions (neural clusters around EEG channels) and at different times during the STDP learning process with EEG data. In each cluster, the spike events correspond to significant changes in the values of PSP pattern that exceed the spike-emitting-threshold. This allows to investigate which areas of the brain were activated at what time for control vs. OP groups. The red boxes illustrate the spike-event actions, described in Section 3.4.

The spatiotemporal rules lead to improve the explainability of the SNN models of brain data and the underpinning cognitive functions. To detect the spatiotemporal spike events in each dynamic cluster, we applied a spike-emitting threshold ℓ to the PSP patterns (plotted in Figure 8). If the PSP pattern of cluster i at time t exceeds the ℓ threshold, then this cluster is recognized as an activated cluster and produces a spike at t. This is applied to all the PSP patterns of 26 clusters for both control and OP groups (depicted in Figure 14). This resulted in forming sequences of spike events that occurred at a certain spatial position (neural cluster corresponds to specific EEG electrode) at different time points. The occurrence of spike events in different classes can be defined by spatiotemporal rules to explain the difference in the interactions between EEG channels.

As seen in Figure 14, the extracted patterns/events from the SNN improve the model explainability by demonstrating where (space) and when (time) a trajectory of frequent behaviors (spike-event actions) take place in the models of brain data from the addictive group versus the control group. Such spatiotemporal patterns may occur in distinct brain regions at certain times, and they can be represented as a set of spatiotemporal rules. The knowledge extracted by the OP group can be compared with control group to reveal the affected brain areas and functions by addiction. For example, it can be seen from Figure 14 that the SNN models produced a greater number of spike-event actions (shown in red boxes) over time in several spatial positions including FP2, F3, F7, and Oz in OP group than the control group. Two symbolic representations of the rules for control group ($R_{1})$ and OP group ($R_{2})$ are defined as follows, where ordi (i=1,2,…) defines the order of the time interval when maximum events are detected:

$$R_{1}:IF\;{\left\{CP4,ord_{1}\right\}}_{\;}AND\;\left\{T3\;,\;ord_{2}\right\}$$

$$AND{\left\{Cz,ord_{3}\right\}}_{\;}AND\;\left\{Fp2\;,\;ord_{4},\;ord_{5}\right\}$$

$$AND\;\left\{Fpz\;,\;ord_{6}\right\}$$

THEN Output =1

$$R_{2}:IF\;{\left\{Oz,ord_{1}\right\}}_{\;}AND\;\left\{Cp4\;,\;ord_{2}\right\}\;\;\;\;\;\;$$

$$AND{\left\{Oz,ord_{3}\right\}}_{\;}AND\;\left\{Fp2\;F3\;F7\;Cpz\;O2,ord_{4}\right\}$$

$$\mathrm{AND}{\left\{Oz,ord_{5}\right\}}_{\;}AND\;\left\{F3\;Cz\;T4,ord_{6}\right\}$$

$$\mathrm{AND}{\left\{O2,ord_{7}\right\}}_{\;}\mathrm{AND}\;\left\{F7\;,ord_{8}\right\}$$

$$\mathrm{AND}{\left\{Fp2,ord_{9}\right\}}_{\;}\mathrm{AND}\;\left\{Fp2\;F3\;,ord_{10}\right\}$$

$$\mathrm{AND}{\left\{F3,ord_{11}\;ord_{12}\;ord_{13}\right\}}_{\;}\mathrm{AND}\;\left\{Fp2\;,ord_{14}\right\}$$

THEN Output =2

## 4. Conclusions and Future Directions

The paper proposes a methodology for deep learning of dynamic spatiotemporal pattern and knowledge discovery and improved explainability of spiking neural networks by modelling the dynamic patterns created during unsupervised learning with streaming spatiotemporal EEG data. The methodology, applied on a BI-SNN architecture exemplified by NeuCube [18], includes procedures for: (1) encoding of the spatiotemporal streaming data into spike sequences; (2) unsupervised learning of the spike sequences in a 3D SNN architecture by creating connections between the neurons; (3) creating dynamic evolving clusters of neurons around the input neurons based on the neuronal spiking activities; (4) continuous validity measurement of the spatiotemporal clusters over the time of their evolution; (5) dynamic visualization of the evolving clusters over time; (6) dynamic feature evaluation; (7) quantitative analysis of the SNN learning patterns; (8) improved classification accuracy, (9) fuzzy clusters, and (10) spatiotemporal rule extractions in SNN model.

In this research, the methodology was illustrated on EEG data of two classes of human subjects in relation to their history of substance use. An assessment of the spatiotemporal clustering patterns of EEG data has led to the detection of important discriminative EEG features in the SNN models. Hence, using only the selected features (by the proposed clustering method) for a classification task, an average of 10% increase in accuracy has been achieved. The clustering approach allowed the learning patterns in the recurrent SNN models to be scrutinized. The findings demonstrate that SNN models are no longer acting as black-box information processing systems. The proposed system is a generic cognitive data analytics framework, applicable to various spatiotemporal data including brain data, and offers a better understanding of the dynamics of streaming data as well as explainability of the models.

For further development of the proposed clustering approach, we aim to enhance it towards early prediction of patterns during unsupervised learning in SNN models. To this aim, the dynamics of the SNN clusters need to be mathematically modelled using differential equations. Consequently, using only a spatiotemporal chunk of streaming data, the next sequential activated areas in the SNN models can be potentially predicted by the proposed clustering technique. This method also needs to be generalized for other types of spatiotemporal data, including environmental data, seismic data, and so forth. The proposed spatiotemporal rules extracted from the dynamic clustering patterns need to be further studied to identify the importance of different areas of neurons in SNN [18,20]. This can be used to detect abstractions from SNN models for a further development of deep learning in SNN architecture. Therefore, the achieved knowledge discovery in SNN models is a significant contribution to explainable machine learning and open AI systems.

The proposed clustering method is a generic approach, tested in this study on an EEG dataset as an example, but this can be applied to any kind of spatiotemporal brain data to extract rules in relation to different cognitive states, such as depression, dementia, and stroke.

## Figures and Tables

**Table 1:** A *t*-test measure was applied to the $P_{max}$ (left), the area under the curve of PSP (middle) and the midrange of the PSP (right) to identify how two classes control and OP are statistically significant. Among these eight top EEG channels, channel 17 has the lowest p-value, representing the highest discriminative power between the samples from different classes.

$P_{max}$			Area under Curve			Midrange of the PSP		
p-Value	EEG Channel	Channel Index	p-Value	EEG Channel	Channel Index	p-Value	EEG Channel	Channel Index
2.4 × 10^−11^	CPz	17	1.2 × 10^−11^	CPz	17	−1 × 10^−11^	CPz	17
2.2 × 10^−9^	C4	14	1.3 × 10^−8^	C4	14	8.4 × 10^−9^	C4	14
4.7 × 10^−9^	Pz	21	2.4 × 10^−8^	P4	22	1.7 × 10^−8^	Pz	21
9.9 × 10^−9^	P4	22	1.8 × 10^−7^	Pz	21	4.9 × 10^−8^	P4	22
0.00001	F4	6	7.3 × 10^−6^	F4	6	2.2 × 10^−6^	F4	6
0.00008	C3	12	3.9 × 10^−5^	C3	12	8.2 × 10^−5^	C3	12
0.00008	Fz	5	0.0007	T6	23	0.0001	Fz	5
0.0002	T6	23	0.002	Fz	5	0.0003	T6	23

**Table 2:** The classification accuracy between EEG samples in control and OP obtained when using all EEG variables versus using the eight top-informative variables selected with the use of the proposed dynamic spatiotemporal clustering method.

Methods	SNN	SVM	MLP	MLR	ECM
26 variables (reported in [33])	85.00	68.00	78.00	68.00	70.00
8 selected variables (feature selection)	92.00	70.00	80.00	72.00	78.00

## Data Availability

The data presented in this study are available on request from the corresponding author. The data are not publicly available due to the terms of consent for research participation stipulate that an individual’s data can only be shared outside of the investigators group if the group has reviewed and approved the proposed secondary use of the data.
